# Exercise Therapy for Chronic ECU Tenosynovitis: A Case Report

**DOI:** 10.3390/reports9020157

**Published:** 2026-05-19

**Authors:** Elena Lanfranchi, Roberto Tedeschi, Milva Battaglia

**Affiliations:** 1 Independent Researcher, 40100 Bologna, Italy; 2 Department of Medicine and Health Science “Vincenzo Tiberio”, University of Molise, “Cardarelli Hospital”, 86100 Campobasso, Italy; 3 Independent Researcher, 40100 Bologna, Italy; 4 Diagnostic and Interventional Radiology, IRCCS Istituto Ortopedico Rizzoli, 40136 Bologna, Italy

**Keywords:** ECU tenosynovitis, extensor carpi ulnaris, wrist rehabilitation, exercise therapy, conservative management

## Abstract

**Background and Clinical Significance**: This case highlights the management of chronic extensor carpi ulnaris (ECU) tenosynovitis in a patient exposed to non-traditional wrist-loading activities. Exercise therapy rehabilitation is well established in shoulder and knee tendinopathies, although it remains less well described for wrist tendinopathies beyond De Quervain’s disease. Moreover, the patient’s active engagement in non-traditional, wrist-intensive sports such as handstands, slacklining, and yoga may have contributed to the development and persistence of chronic extensor carpi ulnaris (ECU) tenosynovitis. Unlike more common ECU injuries observed in tennis or golf players, this case demonstrates how ECU tenosynovitis can develop in less conventional sports. It adds to the scientific literature by showing that chronic ECU tenosynovitis can be effectively managed through non-surgical rehabilitation tailored to the specific needs of the patient, in particular by using exercise therapy. **Case Presentation:** The patient presented with chronic left wrist pain, especially during ulnar deviation and resisted ECU testing, following two traumatic events. Examination revealed limited range of motion caused by pain, particularly in flexion, extension, and both ulnar and radial deviations. Ultrasound imaging confirmed ECU tenosynovitis with mild inflammation of other wrist tendons and a small synovial cyst on radio-scapho-lunate level. ECU stability during forearm rotation was confirmed both clinically and by ultrasound.  The diagnosis of chronic ECU tenosynovitis was managed conservatively with a targeted rehabilitation program focused on isometric strengthening and progressive resistance exercises. Over one month, the patient demonstrated marked improvement in wrist strength, pain reduction, and functional capacity, allowing for a gradual return to sporting activities. **Conclusions:** The main takeaway from this case is that chronic ECU tenosynovitis can be successfully managed through individualized, conservative treatment based on exercise therapy. Early intervention, patient adherence, and rehabilitation tailored to the athlete’s specific demands are crucial for recovery, even in chronic cases, without the need for surgical intervention.

## 1. Introduction and Clinical Significance

Injury to the extensor carpi ulnaris (ECU) tendon is a recognized condition among athletes involved in activities requiring repetitive wrist extension and ulnar deviation. The ECU tendon plays a crucial role in stabilizing the ulnar side of the wrist during rotational movements, especially under load [1,2]. As a result, sports that demand intense and repetitive wrist actions—such as gymnastics, yoga, climbing, and slacklining—are associated with an increased risk of overuse injuries to this structure [3,4]. Tenosynovitis, an inflammatory condition of the tendon sheath, occurs when excessive stress leads to inflammation, thickening of the synovial lining, and fluid accumulation, often resulting in pain, weakness, and functional limitations [5].

The ECU tendon is particularly vulnerable in athletes due to its unique anatomical position, running within a fibrous sheath along the ulnar side of the wrist [1]. This sheath is critical for keeping the tendon in place during motion. Physiologically, it is located more dorsally when the forearm is supinated, and more ulnarly when it is pronated, increasing in one case the stability of radio-ulnar joint and in the other its contribution to ulnar deviation [2,6,7]. In cases of overuse or trauma, as in the current case, tenosynovitis may develop, potentially leading to tendon instability, subluxation, or even rupture in severe cases. Early diagnosis and appropriate management are essential to prevent progression to chronicity and more significant structural damage.

Conservative treatment, including rest, immobilization, and rehabilitation exercises, is typically the first line of therapy for ECU tenosynovitis [6]. The goal is to reduce inflammation, restore normal tendon function, and gradually reintroduce the athlete to their activities. Rehabilitation protocols often focus on isometric exercises, eccentric strengthening, and proprioceptive training to stabilize the wrist and prevent recurrence [8]. In refractory cases or when instability is present, corticosteroid injections and surgical intervention may be considered. However, in this case, the absence of ECU instability and the patient’s compliance with a structured rehabilitation plan allowed for a successful conservative approach, underscoring the potential for non-invasive management of chronic tendinopathies.

## 2. Case Presentations

This case involves a 33-year-old male, highly active in sports such as handstands, slacklining, yoga, and occasional climbing, who presents with chronic left wrist pain. His occupation as a handstand instructor and participation in physically demanding activities put significant stress on his wrists, particularly the non-dominant left one, which became symptomatic after two separate traumas.

The first injury occurred in January 2024 during a slacklining session, where he fell from about one meter, landing on his left wrist. He reported intense pain, rated 9/10, but initial radiographs taken at the hospital showed no fractures or other significant findings. Following the first injury in January 2024, the patient managed the condition autonomously, utilizing rest, ice, and a wrist brace for three days. The patient did not report regular or long-term use of medications at the time of the physiotherapy evaluation. However, he reported occasional use of non-steroidal anti-inflammatory drugs (NSAIDs), specifically ibuprofen, for short-term symptom relief. Although non-steroidal anti-inflammatory drugs (NSAIDs) are sometimes used for short-term symptom control in tendinopathy, prolonged pharmacological management was not considered necessary in this case. Current rehabilitation strategies in tendon disorders generally favour load management and progressive exercise rather than long-term reliance on anti-inflammatory medication. This self-initiated conservative approach led to some symptom relief but did not result in full recovery. The patient did not seek professional medical advice or intervention at this stage. Two months before the most recent evaluation, the patient experienced a second injury while lifting a heavy barrier, which exacerbated the wrist pain and led to constant discomfort, particularly during sports and physical activities.

Clinically, the patient presented with painful limited active wrist motion, particularly in flexion-extension and ulnar-radial deviation. Active range of motion tests revealed pain during passive extension and ulnar deviation, with subjective weakness and fatigue in the left wrist. Special tests for Triangular Fibrocartilage Complex (TFCC) instability, such as the fovea and ballottement tests, were negative, but the ECU synergy test was positive for pain without signs of subluxation, leading to the hypothesis of ECU tenosynovitis. Despite some mechanical limitations, the patient demonstrated preserved wrist strength on manual testing, albeit with some discomfort during ECU resistance, assessed through a Neuromuscular Cranio-cervical Device (NOD) dynamometer (OT Bioelettronica, Turin, Italy, v 1.3).

An ultrasound [9] of the left wrist, performed in September 2024, revealed chronic reactivation of ECU tenosynovitis, with thickening of the synovial sheath and mild hyperemia, indicative of ongoing inflammation. No dynamic instability was detected, which is noteworthy in a case of overuse injury of this tendon. Additional findings included mild fluid tenosynovitis in the extensor carpi longus et brevis radialis (ERBC and ERLC) and extensor pollicis longus (EPL), as well as a small 3 × 3 mm^2^ synovial cyst at the radioscapholunate junction, consistent with repetitive microtrauma. No significant abnormalities were observed in the flexor tendons.

This case is notable for the chronicity of symptoms despite the absence of significant structural damage on initial imaging. The patient’s physical demands, coupled with his intermittent use of protective measures like wrist braces and continued engagement in sports without a specific protocol for load management through specific rehabilitation exercise therapy, likely contributed to the persistence of symptoms. The diagnosis [10] of ECU tenosynovitis, in the absence of tendon instability, is somewhat underdescribed in scientific literature, where dynamic subluxation or rupture is often observed in chronic cases.

Treatment involved a structured home-based rehabilitation program, focused on isometric strengthening of the ECU and progressive load-bearing activities to enhance wrist stability. The exercises targeted ECU strength through resistance band work, isometric contractions, and proprioceptive drills, tailored to his unique sporting needs. The patient demonstrated good adherence to the program after the first week, as he initially experienced chest pain that prevented him from performing wrist exercises. Initially he performed daily exercises and reported significant improvements in pain and function after three weeks. The physical therapist had in mind to check the patient after 2 weeks to improve the exercises, but he presented only 2 weeks later since he did not perform the exercise in the first week due to chest pain and subsequently had a busy work schedule. By early October 2024, the patient had regained considerable wrist strength, nearly matching that of the unaffected side, with only mild discomfort during testing.

The patient’s ability to return to his handstand practice, albeit in a limited capacity, reflects the success of the conservative management plan. However, he remains cautious with certain yoga positions that place excessive strain on the wrist, modifying his activities to minimize the risk of recurrence in yoga, giving priority to hand standing. His compliance with the rehabilitation protocol (after the first week) and gradual reintroduction of sports-specific activities, such as handstands and slacklining, have been key factors in his recovery.

This case underscores the importance of early diagnosis and intervention in chronic tenosynovitis of the wrist, particularly in athletes or individuals with high physical demands. The absence of ECU instability, despite chronic inflammation, and the presence of a synovial cyst highlight the complexity of managing overuse injuries in sports-related contexts. A conservative approach, with a focus on strengthening, proprioception, and load management, proved effective in this case, allowing the patient to return to near full function without the need for surgical intervention. This case illustrates the delicate balance between rest and activity that is critical in managing chronic musculoskeletal conditions in athletes and physically active individuals.

### 2.1. Clinical Findings

During the physical examination, several significant findings were noted, particularly in relation to the left wrist, which had sustained multiple traumas. The patient demonstrated limitations in active range of motion (AROM), particularly in wrist flexion and extension. AROM revealed flexion of 90° and extension of 74°, with passive motion being full (90/90°) but eliciting pain, particularly at the end range of extension release (NPRS 7/10). Notably, ulnar and radial deviation were markedly reduced, with active ranges of 30° for both directions (compared to the contralateral wrist, which had 40°). During active ulnar deviation, the patient experienced significant pain (NPRS 8/10), while radial deviation caused moderate discomfort (NPRS 5/10). Passive ulnar and radial deviations were symmetrical bilaterally at 50°, but caused intense fatigue sensations in the left wrist.

A brief upper limb screening examination was also performed. Elbow range of motion was full and pain-free. Sensory screening using light touch testing did not reveal abnormalities, and motor testing of the wrist and hand muscles was preserved. These findings supported the interpretation that the patient’s symptoms originated locally from the wrist rather than from proximal neurological sources.

Pronation and supination movements were complete; however, the patient reported pain localized along the extensor carpi ulnaris (ECU) during supination. Strength testing with a hand dynamometer revealed diminished force in the left wrist, particularly when compared to the unaffected side. Resistance testing of the ECU tendon produced notable discomfort, reproducing the patient’s familiar pain (Table 1).

Several special tests were conducted to assess the integrity of the wrist structures. The fovea test (palpation of the triangular fibrocartilage complex [TFCC]), ulnar ballottement, and lunate ballottement tests were all negative, indicating no obvious ligamentous instability or TFCC tear. Additionally, considering the patient’s history of a fall on an outstretched hand, specific tests for scapholunate ligament integrity were performed. The Watson test, SL ballottement, and axial compression of the thumb were all negative, confirming the absence of pain or instability in that region. It is worth noting that although a small synovial cyst was observed in the radioscapholunate area on imaging, this finding was not correlated with the patient’s symptoms. The ECU synergy test, which assesses for ECU tendon instability, was positive for pain but did not reveal subluxation of the tendon. This result suggests the presence of an inflammatory process rather than mechanical dysfunction of the ECU. Additionally, resisted ECU testing exacerbated the pain, further confirming the involvement of this tendon in the patient’s symptoms.

Overall, these clinical findings point toward a diagnosis of chronic ECU tenosynovitis with functional impairment, likely exacerbated by the patient’s continued engagement in sports that demand high wrist stability, such as handstands, slacklining, and yoga. The absence of dynamic instability in the ECU, despite significant pain and dysfunction, adds a layer of complexity to the case, suggesting that the pathology is primarily inflammatory rather than mechanical.

### 2.2. Timeline

January 2024: Patient sustains a fall from 1 m while slacklining, landing on the non-dominant left wrist. Immediate pain (9/10). Radiographs at S. Orsola Hospital show no fractures. Initial management includes rest, ice, and a wrist brace for three days.

Mid-2024: The patient reports persistent wrist pain despite initial improvement, worsened by lifting a barrier, leading to chronic discomfort.

September 2024: Physical examination reveals limitations in wrist movement and pain with ulnar deviation. Ultrasound confirms chronic ECU tenosynovitis, mild fluid tenosynovitis of ERC and EPL, and a small synovial cyst (3 × 3 mm).

September to October 2024: The patient begins a structured rehabilitation program, focusing on isometric ECU strengthening. Improvement is noted in strength and pain reduction after one month, allowing a partial return to sports activities.

October 2024: Follow-up shows significant recovery, with nearly symmetrical wrist strength and reduced pain, though the patient remains cautious during certain activities.

### 2.3. Diagnostic Assessment

The diagnostic process for this patient included a thorough physical examination and imaging studies. Initial radiographs performed after the first traumatic episode (approximately nine months before the physiotherapy evaluation) did not reveal fractures, carpal instability, or relevant anatomical abnormalities. In particular, no abnormal ulnar variance was reported. Given the absence of mechanical instability, neurological symptoms, significant loss of strength, or clinical suspicion of major structural lesions, ultrasound imaging was considered sufficient for diagnosis and follow-up in this case. Ultrasound allowed dynamic assessment of the ECU tendon during forearm rotation, confirmed the absence of subluxation, and demonstrated active inflammatory findings including synovial sheath thickening and hypervascularisation. Furthermore, the favourable clinical progression during rehabilitation reduced the indication for MRI investigation, which was considered unnecessary in the absence of persistent diagnostic uncertainty or surgical planning.

The physical examination revealed significant limitations in wrist range of motion, particularly in ulnar deviation, along with pain during resisted movements, specifically involving the extensor carpi ulnaris (ECU). Special tests, such as the fovea test and ECU synergy test, helped rule out TFCC involvement and ECU tendon subluxation, while confirming the presence of pain related to ECU tenosynovitis.

**Figure 1:**
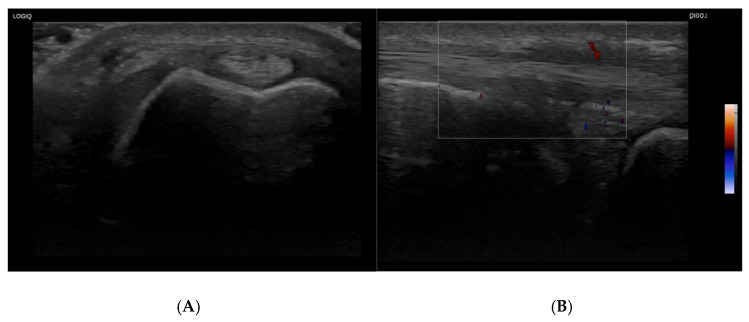
Ultrasound findings of chronic extensor carpi ulnaris (ECU) tenosynovitis. Ultrasound imaging of the extensor carpi ulnaris (ECU) tendon. (**A**) Transverse view showing synovial sheath thickening consistent with chronic tenosynovitis. (**B**) Longitudinal view with colour Doppler demonstrating mild ypervascularization, indicative of active inflammatory changes. No evidence of dynamic instability is observed.

Diagnostic imaging, specifically an ultrasound performed in September 2024, confirmed the clinical suspicion of chronic ECU tenosynovitis. The ultrasound showed thickening (Figure 1) of the ECU synovial sheath, mild hyperemia, and no dynamic instability (Supplementary File). Additionally, fluid tenosynovitis was detected in the extensor carpi radialis communis (ERC) and extensor pollicis longus (EPL), with a small 3 × 3 mm synovial cyst at the radioscapholunate junction, indicating underlying degenerative changes.

There were no significant diagnostic challenges, as the patient had timely access to imaging and physical assessments, and no financial or cultural barriers were reported. The primary diagnosis of chronic ECU tenosynovitis was confirmed, with other differential diagnoses like TFCC tears and ECU subluxation effectively ruled out. The prognosis is favorable given the positive response to conservative treatment, with a gradual return to functional activities and sports anticipated within the coming months.

### 2.4. Therapeutic Intervention

The therapeutic approach for this patient primarily involved conservative management, focusing on a comprehensive assessment, load management rehabilitation and self-care strategies (Table 1). Given the nature of the injury—a chronic reactivation of extensor carpi ulnaris (ECU) tenosynovitis without dynamic instability—the primary therapeutic interventions were non-pharmacologic and aimed at reducing inflammation and load management, improving tendon strength, and restoring function.

#### 2.4.1. Pharmacologic Intervention

Before presenting to physical therapy, the patient managed acute pain episodes with nonsteroidal anti-inflammatory drugs (NSAIDs), specifically ibuprofen (brand: Moment), to alleviate inflammation and provide short-term relief, particularly during a recent episode of costal pain. Although the medication provided temporary symptom relief, it was not a long-term solution due to its limited effect on the chronic tenosynovitis of the wrist. No specific dosage or long-term NSAID regimen was prescribed for the wrist condition, as the focus shifted toward non-pharmacologic interventions.

#### 2.4.2. Rehabilitation and Self-Care

**Figure 2:**
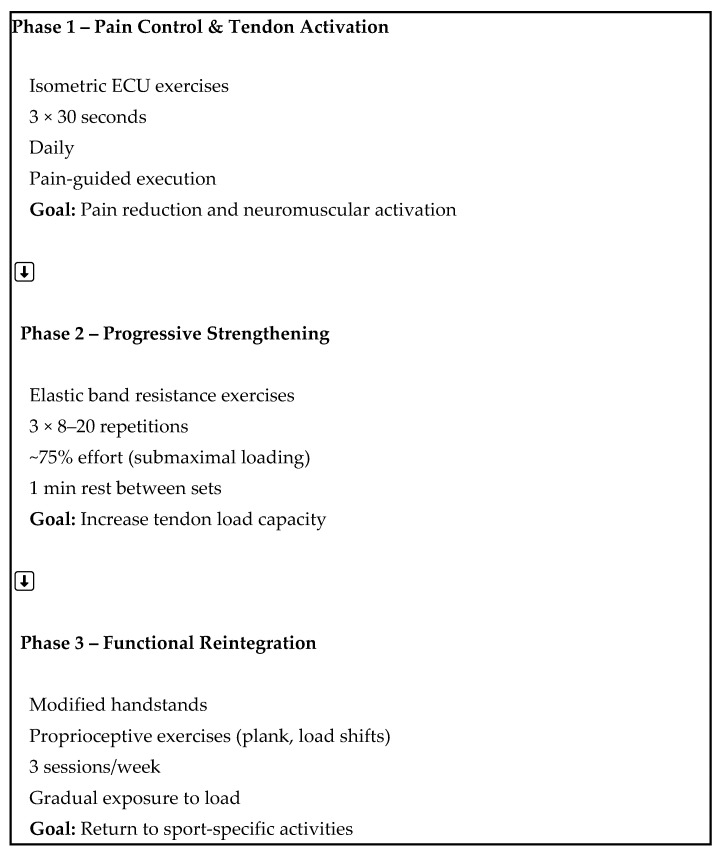
Schematic representation of the rehabilitation program.

A home-based exercise-based rehabilitation program (Table 1, Supplementary File and Figure 2) was the cornerstone of the therapeutic intervention. The program was designed to strengthen the ECU tendon and improve wrist stability, while gradually reintroducing the patient to functional activities. The rehabilitation approach was consistent with contemporary tendon-loading principles described in the literature, emphasizing progressive loading, symptom monitoring, and gradual return to functional activities [11,12,13]. The loading strategy was selected according to contemporary tendon rehabilitation principles suggesting that submaximal progressive loading may improve tendon load tolerance, neuromuscular control, and pain modulation while minimizing excessive symptom irritability during the early stages of rehabilitation [14]. A schematic representation of the rehabilitation program is provided in Figure 2 to improve clarity and reproducibility.

The specific components of the rehabilitation plan included:

To ensure full consistency and reproducibility, the rehabilitation protocol was standardised across all sections of the manuscript (text, table, and schematic diagram).

Phase 1 (Pain Control and Activation) [15]: The patient performed isometric ECU exercises daily, consisting of 3 sets of 30-s holds in a pain-free range. The primary goal was pain reduction and early neuromuscular activation.
Phase 2 (Progressive Strengthening): Elastic resistance exercises were introduced, consisting of 3 × 8–12 repetitions performed at approximately 75% of perceived maximal effort, corresponding to a submaximal intensity that allowed the patient to complete each set with moderate fatigue while maintaining proper movement quality and pain levels below 3/10 on the NPRS. The load was progressively increased up to 15–20 repetitions based on tolerance.
Phase 3 (Functional Reintegration) [4]: Functional and sport-specific exercises (e.g., modified handstands and proprioceptive loading tasks) were performed three times per week, with gradual exposure to increasing wrist load.

Progression criteria were based on pain monitoring, exercise tolerance, and functional capacity rather than fixed timelines. Exercise progression was permitted when the patient could complete the prescribed exercises without symptom exacerbation during the following 24 h, following a symptom-monitoring approach commonly used in tendon rehabilitation [13]. Load progression was achieved by increasing repetitions, time under tension, resistance, and sport-specific wrist loading demands. This approach was consistent with contemporary evidence suggesting that progressive tendon loading should be individualized according to symptom irritability and functional tolerance rather than based exclusively on predetermined timelines [14,16].

#### 2.4.3. Changes in Therapeutic Intervention

The rehabilitation plan was supposed to be adjusted after 2 weeks, but the patient presented only after a month. Initially, the patient was advised to perform daily exercises for 2 weeks, but due to costal pain from a separate injury, he started it a week later and his routine was modified. In the second and third weeks, the patient adhered well to the daily exercise regimen, but in the fourth week, exercise frequency was reduced to three times per week due to symptom improvement and work-related commitments. Despite this reduction, the patient continued to improve, reporting increased wrist strength and confidence in functional use.

At the one-month follow-up in October 2024, the patient’s therapeutic regimen was revised to further progress his recovery:

- Isometric Exercises were modified to increase the difficulty by changing the hand position: instead of resting the wrist in a neutral position, the patient performed the exercises with the wrist extended and the hand pressing down on the table, simulating the stress experienced during handstands.
- The patient was also instructed to add subtle dynamic movements during isometric holds (shifting weight slightly forward, backward, left, and right) to more closely mimic the demands of his handstand practice. This addition aimed to further challenge the ECU tendon while ensuring controlled progression.
- Resistance Training with the elastic band was maintained, but the patient was encouraged to progressively increase repetitions and sustain each contraction for 10 s by the eighth repetition. This change was intended to increase tendon endurance while preparing the patient for a full return to physical activity.

Given the patient’s progress and reduced pain levels, the frequency of formal follow-up sessions was reduced, with more emphasis placed on self-monitoring and continuation of the home exercise program. The patient remained compliant, reporting substantial improvements in wrist stability, grip strength, and confidence in performing handstands. The long-term goal remained to fully return the patient to his pre-injury activity level, including teaching handstands, without restrictions.

#### 2.4.4. Preventive Measures

Preventive strategies were also emphasized, given the repetitive nature of the patient’s sports and occupational activities. These included continued use of the wrist brace during high-impact activities (e.g., climbing) and education on proper wrist positioning during handstands to avoid excessive ulnar deviation or hyperextension. Additionally, the patient was encouraged to integrate regular rest periods into his routine to prevent overuse injuries, especially given the risk of recurrence in such physically demanding practices.

In conclusion, the therapeutic intervention for this patient was primarily focused on conservative, non-surgical management with a heavy emphasis on rehabilitation exercises and self-care. Adjustments to the rehabilitation plan were made based on the patient’s progress and symptomatology, with the goal of restoring full function and preventing future injury recurrence.

Supplementary File Demonstration of the Progressive Resistance Training Exercises for ECU Tenosynovitis Rehabilitation.

A transverse and B longitudinal L8-18i Mhz US images of the extensor carpi ulnaris reveal chronic hypertrophic tenosynovitis with a mild hypervascularization on color Doppler.

Images illustrating the progressive resistance training exercises prescribed for the rehabilitation of chronic ECU tenosynovitis. The exercises involve the use of elastic bands to perform concentric, eccentric, and isometric movements targeting the extensor carpi ulnaris tendon. These exercises were performed in a seated position to ensure stability and focus on controlled wrist movements. The protocol included 3 sets of 10 repetitions at 75% perceived maximal effort, progressing to 3 sets of 20 repetitions with 20-s holds.

#### 2.4.5. Follow-Up

Follow-up assessments were conducted approximately one month after the initiation of the rehabilitation program, with an additional clinical reassessment performed in November 2024. During follow-up, both clinician and patient-assessed outcomes were quantified using strength and pain scales. By October 2024, grip strength measurements showed significant improvement compared to the initial evaluation. On neutral pronation, the grip strength increased from 6–8 kg in the left wrist to 11 kg (matching the right wrist). For pure pronation, the patient’s strength improved from 11 kg to 13 kg on both sides, with minimal discomfort (NPRS 1/10 during the second test). These repeated evaluations allowed monitoring of the progression of strength recovery and symptom reduction over time.

During more strenuous activities, such as standing ECU testing, the patient showed a stable left wrist strength ranging from 17 to 18 kg, compared to an initial 6–18 kg range, with a pain score of NPRS 2/10 during the first test and NPRS 4/10 during the second test. The ECU-specific strength progressed from 9 kg initially to 12 kg after one month of rehabilitation. These metrics demonstrated near-symmetry with the unaffected wrist, which recorded similar values (Table 2). A retrospective Quick Disabilities of the Arm, Shoulder and Hand questionnaire (QuickDASH) assessment was also obtained to provide an additional patient-reported functional perspective. The QuickDASH score improved from approximately 66/100 before rehabilitation to 0/100 at the most recent reassessment, reflecting substantial reduction in perceived disability. The main pre-rehabilitation limitations involved sport-specific wrist loading, heavy upper-limb tasks, pain during functional activities, and work-related wrist demands, all of which were reported as absent or minimal at the latest evaluation.

Adherence to the exercise program was high, with the patient following the prescribed exercises daily for the first two weeks and reducing frequency to three times per week thereafter due to external costal pain. This minor adjustment did not hinder overall progress, as evidenced by the improved strength and reduced pain levels. Importantly, no adverse events related to the rehabilitation process were reported, and the patient tolerated the interventions well without significant exacerbations or setbacks. At the follow-up on 20 November 2024, further improvements in wrist strength and functionality were recorded. The grip strength [17] was measured using a NOD dynamometer, showing right/left maximal values of 12.5/13 kg in a neutral position (NPRS 1) and 14/14 kg in pronation (NPRS 1 during the second test). During standing ECU testing, the average left wrist strength reached 24 kg (28 + 24 + 22 = 24.6 kg on the right vs. 23 + 24 + 25 = 24 kg on the left; NPRS 1). Specific ECU strength was recorded at 14/14 kg (NPRS 1 during the second test). These metrics demonstrate continued near-symmetry between wrists, reflecting the effectiveness of the rehabilitation protocol. The patient continues exercises every other day, performing 3 sets of 10 repetitions with an elastic band at 75% perceived maximal effort, progressively increasing to 3 sets of 20 repetitions with a 20-s hold on the last repetition and a 1-min rest between sets.

## 3. Discussion

This case report offers valuable insights into the conservative management of chronic extensor carpi ulnaris (ECU) tenosynovitis in a physically active individual, highlighting both strengths and limitations [1]. One of the key strengths lies in the patient’s clear response to a well-structured rehabilitation program that emphasized progressive loading and isometric strengthening [18]. The use of ultrasound imaging to confirm the clinical diagnosis of ECU tenosynovitis and to rule out more severe conditions like tendon subluxation or TFCC tears strengthened the diagnostic accuracy and guided an appropriate non-surgical treatment pathway. Although the rehabilitation principles applied in this case are consistent with contemporary tendon-loading paradigms commonly used for tendinopathies, the ultrasonographic findings predominantly demonstrated synovial sheath thickening and hypervascularisation rather than clear intratendinous degenerative abnormalities. Therefore, the condition was interpreted clinically as a predominantly inflammatory tenosynovial disorder with possible secondary reactive tendon involvement. The favourable clinical progression further supported the diagnostic hypothesis, reducing the need for additional imaging such as MRI. This approach was particularly relevant given the patient’s specific functional demands. The patient’s adherence to the exercise regimen and the gradual improvements in pain and function demonstrate the efficacy of tailored rehabilitation in resolving chronic tenosynovitis without requiring invasive procedures.

However, there are several limitations to this case. First, the absence of comparative long-term follow-up beyond one month makes it difficult to fully assess the long-term outcomes of the rehabilitation program, especially considering the patient’s high-demand activities. Another limitation concerns the use of the NOD dynamometer for strength assessment. Although a retrospective QuickDASH assessment was obtained to provide additional patient-reported information, the absence of prospectively collected wrist-specific outcome measures remains a limitation. The lack of these measures limits comparability with existing literature and reduces the external validity of the findings, as subjective patient-perceived improvement cannot be directly quantified using standardised tools. Although this device has been used in neuromuscular research, its application for hand strength measurement is less standardized compared to widely validated tools such as the Jamar dynamometer. Therefore, the reported strength values should be interpreted with caution [19,20]. While the short-term results are promising, the risk of recurrence [4], particularly given the patient’s continued participation in activities like handstands and slacklining, remains uncertain. Another limitation is the lack of detailed biomechanical assessments, such as force plate analysis or motion capture, which could have provided a more comprehensive understanding of the wrist’s functional recovery and stability under load. However, maximal load capacity was assessed during rehabilitation, providing a clinically relevant proxy of functional tendon adaptation. Additionally, the absence of pain [21] modulation strategies beyond NSAIDs, such as manual therapy or electrotherapy, might have left potential therapeutic options unexplored. The fact that the rehabilitation plan was adapted to the patient’s costal pain also presents a minor limitation, as the temporary reduction in exercise frequency may have impacted the consistency of strength gains.

The literature on ECU tenosynovitis primarily focuses on athletes [4], particularly those engaged in sports involving repetitive wrist use, such as tennis, golf, and climbing. While the rehabilitation principles applied in this case are consistent with current tendon-loading paradigms, the present report contributes to the literature by extending their application to a less commonly described anatomical region and clinical context. In particular, ECU tenosynovitis associated with non-traditional wrist-loading activities such as handstands and slacklining remains underreported. Therefore, the added value of this case does not lie in introducing novel interventions, but rather in demonstrating the adaptability and effectiveness of established tendon-loading strategies in a highly specific functional scenario. This reinforces the importance of individualised load management in athletes exposed to unconventional biomechanical demands.

Most reports emphasize the importance of early diagnosis to prevent progression to instability, subluxation, or rupture, and typically advocate for conservative management initially. In line with these findings, this case supports the existing literature by demonstrating that structured rehabilitation focusing on tendon strengthening can effectively manage chronic tenosynovitis without surgical intervention. Previous studies have also shown the value of ultrasound in diagnosing ECU pathology [10] and tracking recovery, reinforcing the role of imaging in confirming tendon integrity and ruling out more serious complications. However, the literature regarding ECU injuries in individuals who practice handstands or slacklining is limited, making this case particularly relevant for expanding understanding in less common sports.

Additional conservative strategies such as taping or night splints were considered but not systematically implemented in this case. The patient had already used a night splint independently for approximately one month before seeking physiotherapy, reporting limited symptom improvement. For this reason, prolonged immobilization was not reintroduced during rehabilitation. Taping was considered as a potential adjunct intervention but was not implemented because the patient demonstrated progressive improvement with exercise-based rehabilitation alone. Nutritional interventions were not included, as the clinical presentation suggested a localized overload-related tendon disorder rather than a systemic or metabolic condition [22,23].

Other conservative interventions such as corticosteroid injections or extracorporeal shockwave therapy are typically considered in refractory cases. However, given the positive response to structured exercise therapy in this case, additional interventions were not deemed necessary [24,25].

From a scientific perspective, the conclusions drawn in this case are grounded in both the patient’s clinical presentation and the known pathophysiology of ECU tenosynovitis. The chronic inflammation and thickening of the synovial sheath without dynamic instability suggest that the primary cause was repetitive strain from the patient’s activities. The absence of subluxation or rupture, despite chronicity, is notable and can be explained by the integrity of the surrounding wrist structures, which likely helped maintain tendon stability. The small synovial cyst identified on ultrasound is indicative of underlying degenerative changes, likely due to repeated microtrauma, but it did not appear to significantly impact wrist function or recovery. The patient’s progressive recovery with conservative treatment supports the hypothesis that early and sustained intervention, focusing on strengthening and functional load management, is critical for optimal outcomes in chronic tendon disorders. One important limitation of this case report is the relatively short follow-up period. Although the patient demonstrated clear improvements in pain and strength during the rehabilitation phase, longer follow-up would be necessary to determine the long-term stability of these results and the potential risk of symptom recurrence.

This case report highlights the effectiveness of conservative management for chronic ECU tenosynovitis in an athlete, particularly when rehabilitation is tailored to the demands of the patient’s activities. The primary takeaway is that targeted strengthening exercises and progressive load-bearing, combined with patient adherence, can result in significant improvements in function and pain even in chronic cases. These findings should be interpreted cautiously given the inherent limitations of a single case report and the relatively short follow-up period. However, the relatively short follow-up period represents a major limitation of this report, as tendon adaptation, recurrence risk, and durability of symptom resolution in high-demand athletic activities cannot be adequately determined from short-term observation alone. Despite the favourable short-term outcomes, longer-term follow-up would be required to confirm the durability of these results in high-demand activities. Furthermore, the relatively short follow-up period represents an important limitation, as long-term tendon adaptation and recurrence risk cannot be fully assessed. Future studies should include extended follow-up periods to better evaluate the durability of rehabilitation outcomes in high-demand individuals. From a practical perspective, clinicians managing athletes with ECU disorders should consider that persistent ulnar-sided wrist pain in non-traditional wrist-loading sports may reflect chronic ECU tenosynovitis even in the absence of instability. Dynamic ultrasound assessment, progressive tendon-loading rehabilitation, symptom-guided exercise progression, and sport-specific load management may represent key components for successful conservative treatment in high-demand individuals. Given the inherent limitations of a single-case design, these findings should not be generalized to all ECU disorders but may provide clinically relevant insights for individualized rehabilitation planning in similar high-demand athletic populations.

### Patient Perspective

From the patient’s perspective, the rehabilitation program has allowed him to resume activities such as slacklining, including setting up high lines, without concerns about wrist stability even after falls. He has returned to performing handstands without the need for extensive wrist warm-ups and feels that both wrists are stronger compared to a year ago. However, he notes that skipping the elastic band exercises for more than three consecutive days results in a noticeable decline in wrist comfort. Overall, the patient expresses satisfaction with his ability to perform handstands and other activities freely, crediting the exercises for maintaining wrist strength and stability.

## Figures and Tables

**Table 1:** Rehabilitation Protocol.

Exercise	Details	Progression
Isometric Strengthening	3 × 30-s holds (daily)	Maintain duration, increase load tolerance
Progressive Resistance	3 × 8–12 reps (~75% effort)	Progress to 15–20 reps
Proprioception	Plank-based loading (≥60 s)	Increase duration/load
Functional	Sport-specific drills 3×/week	Gradual exposure

**Table 2:** Strength and Pain Evaluation.

Test	Right (kg)	Left (kg)	Pain (NPRS)
Grip Strength-Neutral	12.5	13.0	1
Grip Strength-Pronation	14.0	14.0	1 (during 2nd test)
Standing ECU Strength (Average)	24.6	24.0	1
ECU-Specific Strength	14.0	14.0	1 (during 2nd test)

## Data Availability

The original data presented in this study are available on reasonable request from the corresponding author. The data are not publicly available due to privacy concerns.

## Supplementary Materials

The following supporting information can be downloaded at: https://www.mdpi.com/article/10.3390/reports9020157/s1, Figure S1:Progressive Rehabilitation Exercises and Ultrasound Findings in Chronic Extensor Carpi Ulnaris Tenosynovitis.
