# Supplementary material for: Exercise Therapy for Chronic ECU Tenosynovitis: A Case Report
Source: Reports (MDPI). 2026 May 19;9(2):157. doi: 10.3390/reports9020157 (PMC13214708; doi:10.3390/reports9020157)
Supplement: Supplementary file 1 [file reports-09-00157-s001.zip › reports-4238455-supplementary.pdf]

T0 (Initial Phase, Home Program - Once a Day)	Execution
**Setup**: Perform isometric contractions by pressing the hand against a table, with the forearm pronated and the wrist in a neutral position.<br>**Execution**: 3 sets of 30 seconds (30 seconds rest between sets).	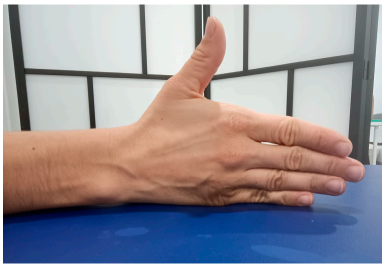
**Elastic Band Exercise**: Perform 3 sets of 12 repetitions of ECU strengthening at approximately 75% of perceived maximal effort, corresponding to a submaximal load performed with moderate fatigue while maintaining movement quality and pain below 3/10 on the NPRS. Rest for 1 minute between sets.<br>**Setup**: Mark the reference point for the full range of motion (e.g., with white tape).<br>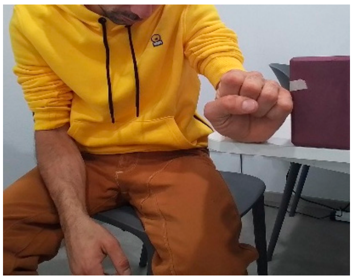	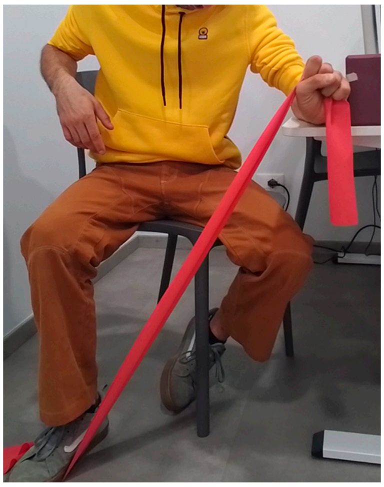
**ECU Isometric with Elastic Band**: Perform with the forearm resting on a table, elbows at 90°, and tension applied between the 3rd and 5th fingers using the elastic band.<br>**Execution**: Hold for isometric contraction.	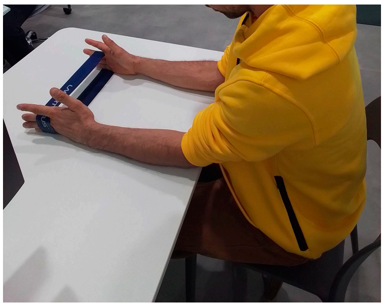
T1 (+4 weeks)	

**Isometric Pressing from Standing Position**: Press the hand against a table with the wrist extended, forearm pronated, and elbow straight. **Execution**: 3 sets of 30 seconds (30 seconds rest). Perform the first set while stationary, and for the next two, shift weight slightly forward/backward and left/right, simulating progressive sport-specific wrist loading during handstand activities.	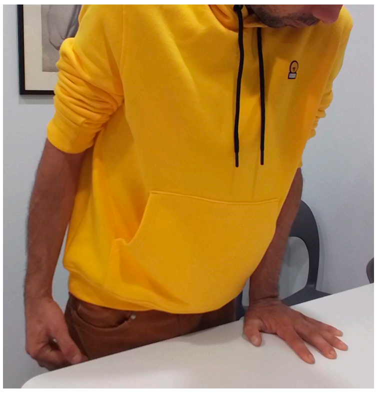
**Elastic Band Exercise**: Perform 3 sets of 8 repetitions of ECU strengthening at approximately 75% of perceived maximal effort (progress to 3 sets of 20 repetitions). **Execution**: Hold the 8th repetition for 10 seconds. Rest for 1 minute.	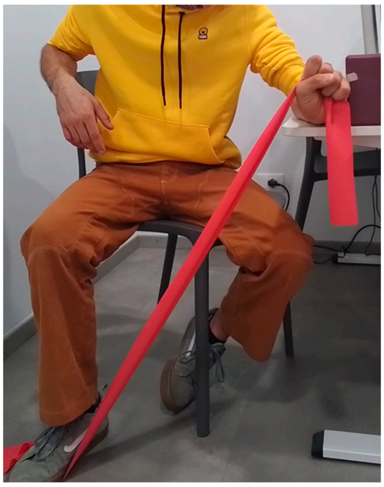
**Proprioceptive Plank Routine**: Perform weight-bearing proprioceptive exercises through the ulnar aspect of the forearm, using an elastic band between the 3rd and 5th fingers. **Execution**: Maintain position for at least 1 minute and 15 seconds.	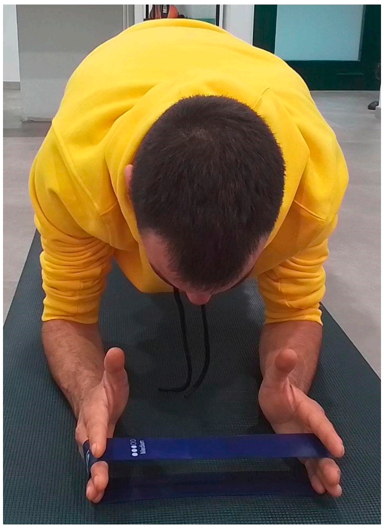
T2 +2 Weeks from T1, +6 Weeks from T0)	

**Advanced Isometric Pressing from Standing Position**: Press the hand against a table with the wrist extended, forearm pronated, and elbow straight. **Execution**: 3 sets of 40 seconds (40 seconds rest). In the first set, stay stationary; for the second and third sets, shift weight slightly forward/backward and left/right. As progression, once you reach 50 seconds, split into two repetitions: the first 25 seconds stationary and 25 seconds with movement, the second entirely with movement.	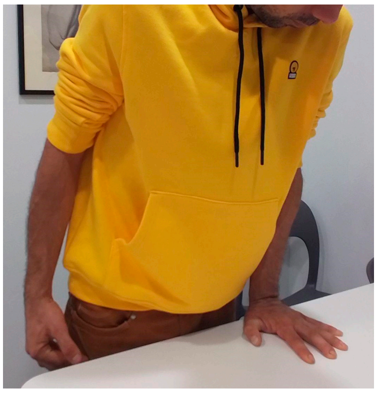
**Elastic Band Exercise**: Perform 3 sets of 10 repetitions of ECU strengthening at approximately 75% of perceived maximal effort (progress to 3 sets of 20 repetitions). **Execution**: Hold the 10th repetition for 15 seconds. Rest for 1 minute (progress to 3 sets of 12 repetitions with 20-second holds). **Progression**: Increased repetitions from 8 to 10, with longer isometric hold times (10 to 15 seconds). Rest increased by 30 seconds to accommodate the added intensity.	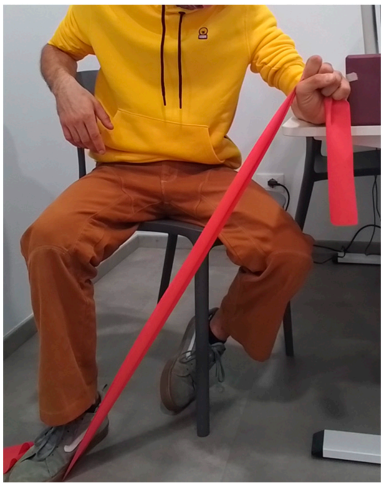

**Proprioceptive Plank Routine**: Perform on the ulnar side of the forearm with an elastic band between the 3rd and 5th fingers.<br>**Execution**: Maintain position for at least 1 minute and 15 seconds (progress to 1 minute and 30 seconds).<br>**Progression**: Increased duration by 15 seconds as it was still challenging, even for the healthy side.	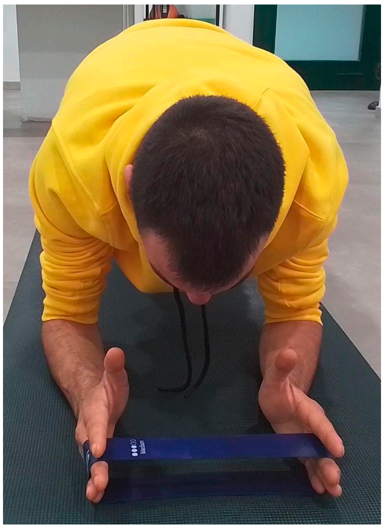

Figure S1. Progressive Rehabilitation Exercises and Ultrasound Findings in Chronic Extensor Carpi Ulnaris Tenosynovitis.
